# Triage accuracy and causes of mistriage using the Korean Triage and Acuity Scale

**DOI:** 10.1371/journal.pone.0216972

**Published:** 2019-09-06

**Authors:** Sun-Hee Moon, Jae Lan Shim, Keun-Sook Park, Chon-Suk Park

**Affiliations:** 1 Department of Nursing, Changwon National University, Changwon, South Korea; 2 Department of Nursing, Dongguk University, Gyeongju, South Korea; 3 Department of Nursing, Chonnam National University Hospital, Gwangju, South Korea; 4 Department of Nursing, Boramae Medical Center, Seoul, South Korea; Technion - Israel Institute of Technology, ISRAEL

## Abstract

**Purpose:**

To identify emergency department triage accuracy using the Korean Triage and Acuity Scale (KTAS) and evaluate the causes of mistriage.

**Methods:**

This cross-sectional retrospective study was based on 1267 systematically selected records of adult patients admitted to two emergency departments between October 2016 and September 2017. Twenty-four variables were assessed, including chief complaints, vital signs according to the initial nursing records, and clinical outcomes. Three triage experts, a certified emergency nurse, a KTAS provider and instructor, and a nurse recommended based on excellent emergency department experience and competence determined the true KTAS. Triage accuracy was evaluated by inter-rater agreement between the expert and emergency nurse KTAS scores. The comments of the experts were analyzed to evaluate the cause of triage error. An independent sample t-test was conducted to compare the number of patient visits per hour in terms of the accuracy and inaccuracy of triage.

**Results:**

Inter-rater reliability between the emergency nurse and the true KTAS score was weighted kappa = .83 and Pearson’s r = .88 (*p* < .001). Of 1267 records, 186 (14.7%) showed some disagreement (under triage = 131, over triage = 55). Causes of mistriage included: error applying the numerical rating scale (n = 64) and misjudgment of the physical symptoms associated with the chief complaint (n = 47). There was no statistically significant difference in the number of patient visits per hour for accurate and inaccurate triage (t = -0.77, *p* = .442).

**Conclusion:**

There was highly agreement between the KTAS scores determined by emergency nurses and those determined by experts. The main cause of mistriage was misapplication of the pain scale to the KTAS algorithm.

## Introduction

As the emergency department (ED) utilization rate increases worldwide, it is imperative to reduce patient overcrowding, to ensure patient safety and to improve the efficiency of emergency care [1]. Triage was introduced to enhance the quality of emergency care by reducing the length of stay of patients and is now applied to EDs [1, 2].

Triage is the process of determining the priority of patient treatment using an internationally recognized 5-level triage system [2]. The best-known triage scales are the Emergency Severity Index (ESI) in the USA, the Canadian Triage and Acuity Scales (CTAS) in Canada, the Australian Triage Scale (ATS) in Australia, and the Manchester Triage Scale in the UK [2,3]. As triage scales should be matched to the specific medical environment in which they are used, the application of a triage scale developed for one country should be approached with caution when applied to another country. Recently, some countries have devised systems that modify major triage scales to suit their medical environment [3]. In Korea, 97.1% of tertiary hospitals and regional emergency medical centers used triage in 2012, along with various tools such as CTAS, ESI, ATS, and a triage scale based on the Korean Emergency Medical Service Act [4]. Substantial communication difficulties among EDs arose owing to variations in triage scales among centers [4]. The need for a standardized triage system has increased and the Korean Triage and Acuity Scales (KTAS) based on the CTAS was developed and has been used nationwide since 2016 [5].

Triage in the ED is the process of rapidly assessing patients, determining who should receive treatment first, and allocating medical resources according to priority [3]. Accurate triage is a way of ensuring patient safety and reducing ED crowding, but mistriage extends length of stay in the ED and increases patient mortality [6,7]. Overtriage is an overestimation of patients with low severity conditions, resulting in increased resource consumption, ED overcrowding, and increased length of stay, ultimately leading to a lack of medical resources in the ED. [8, 9]. Therefore, evaluating the accuracy of triage and assessing the causes of mistriage is essential for improving patient safety and the quality of emergency care.

In studies on triage accuracy, it is important to determine the gold standard. Previous studies have used various methods to measure triage accuracy in the ED. First, assessment of nurse triage accuracy based on clinical scenarios [10] is necessary. The use of scenarios allows for measurement of evaluator competence in terms of providing consistent information to patients. This would help gather information and clearly define the gold standard in the research design process. However, scenario-based research is not a live setting, and therefore, may not adequately reflect the dynamic situation of EDs. Second, sequential evaluation of the same patient by different evaluators in a real-world setting [11, 12] is necessary. Here, the triage score of senior nurses or doctors is used as the gold standard. However, this method lacks the expert verification procedure to determine the gold standard and is not feasible due to patient safety considerations (a second triage will delay treatment) [13]. A third method to evaluate the triage accuracy is the analysis of the medical records. This method has the advantage of assessing the outcome of decisions made in the real ED environment without delaying patient treatment. A limitation of this method is that the evaluator determines the gold standard by analyzing the medical records without direct examination of the patient. In view of the increasing role played by the nursing records in legal disputes [14] and that nurse triage outcomes in Korea are directly related to the cost of emergency medical care [15], the evidence for triage decisions should be clearly documented. Therefore, evaluation of triage accuracy based on the medical records will be useful not only in assessing the accuracy of the classification of the KTAS but also in evaluating the evidence for nurse triaging decisions resulting in mistriage. In a retrospective analysis of medical records, doctors and nurses independently analyzed the triage data, and inter-rater reliability was achieved when the two evaluators reached the same outcome [16]. However, it was not clear whether the two evaluators were triage experts. The evaluation of triage accuracy requires criteria for the selection of triage experts who can evaluate the causes of triage error and the accuracy of the clinical severity classification.

The KTAS has been used in Korea for a relatively short period due to its recent introduction. The studies on KTAS were focused on the validity of the scale and reported that length of stay (LOS) and mortality decreased after applying KTAS [17, 18]; however, limited studies have evaluated the accuracy of KTAS. Recent pilot studies have sought to confirm agreement between nurses and medical students or between nurses using the KTAS [11, 19]. However, the pilot sample size used in these studies was not sufficient and the cause of mistriage was not evaluated. Recently, it was reported that the overestimation of severity according to the pain criteria in KTAS [20]. Pain is an important criterion to assign KTAS level, and it may be careful not to overestimate.

The purpose of this study was to identify the accuracy of triage based on medical records and to evaluate the causes of mistriage. In order to overcome expertise limitations and lack of cause identification for mistriage observed in the previous triage accuracy studies [11, 16, 19], we selected qualified triage experts and evaluated inter-rater agreement between experts. This study will contribute to improving patient safety and the quality of emergency care by identifying triage accuracy and evaluating the causes of mistriage.

## Materials and methods

### Design

This study was a retrospective review of 1267 randomly selected medical records of adult patients admitted to two EDs between October 2016 and September 2017.

### KTAS algorithm

**Fig 1:**
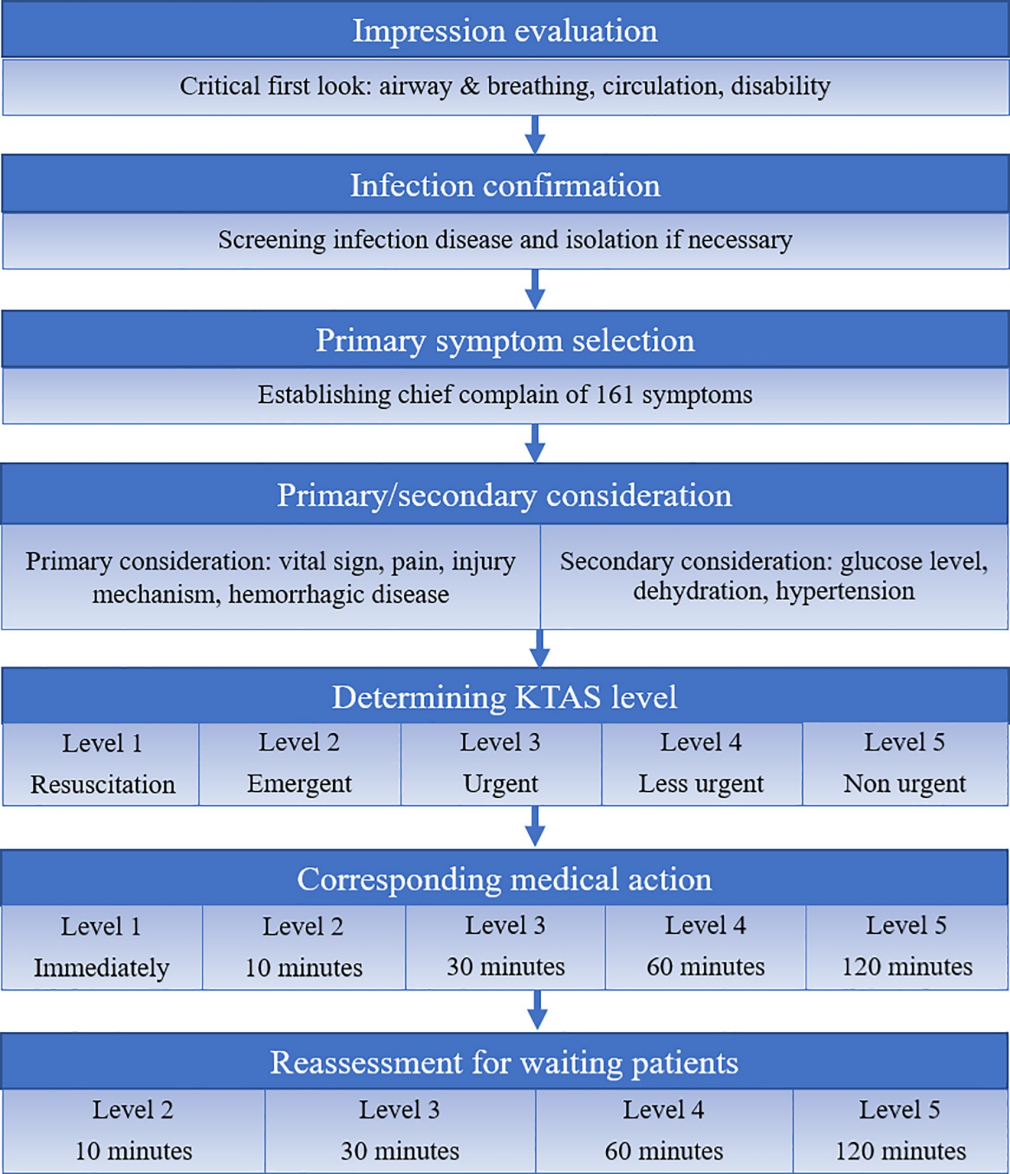
Process of the Korean Triage and Acuity Scales.

KTAS is a five-level triage scale developed in 2012 and based on CTAS, modified to apply to the medical situation in South Korea [5, 15]. KTAS is a symptom-oriented classification tool for evaluating patients. After the critical first-look assessment, emergency nurses determine the triage level by evaluating the primary considerations, including vital signs, pain score, hemorrhagic disease, mechanism of injury, and secondary considerations, including blood glucose level and degree of dehydration (Fig 1) [5, 15]. The KTAS score allocated by the emergency nurses determines patient waiting time until assessment by a ED doctor and the applicable medical treatment fee (emergency, KTAS 1–3 level or non-emergency, KTAS 4–5 level) [5, 15].

### Study process

We investigated the variables based on a literature review as well as previous validity and reliability studies on other emergency triage tools [6, 11, 16, 19]. The measurable variables were then extracted from the medical records. The selected variables were as follows: presenting complaint, current history, KTAS level, demographic data, time of attendance, arrival mode, past history, mental status, vital signs, pain scale, complete time of initial nursing record, and patient disposition.

#### Study setting

We selected 1 regional and 1 local ED based on a number of factors, including patient visits per year, the presence of emergency nurses dedicated to triage only, and collection possibility of the selected variables. Both EDs were academic urban medical centers. The regional ED had approximately 45,000 patient visits per year, and the local ED had approximately 40,000 patient visits per year. Both EDs were divided into a triage area at the entrance and a treatment area. In the regional ED, emergency nurses and doctors took a medical history and performed a physical examination together. The data collected were recorded in the initial nursing record and the doctor’s record, respectively. In the local ED, an emergency nurse conducted a physical examination alone. Initial nursing records from the two EDs included chief complaint, onset time, arrival mode, underlying disease, vital signs, oxygen saturation, surgical history, mental state, pain score, medication and allergy history. The pain scale used in the two EDs was the Numeric Rating Scale (NRS) which consists of a patient self-reporting 11-point scale.

#### Selection of triage experts

We carefully selected experts to determine the triage gold standard outcome. The specific inclusion criteria were as follows: 1) emergency nurses; 2) KTAS instructor and provider; 3) clinical experience in ED of ≥7 years [21]; 4) excellent triage competency. Clinical experience in the ED was defined based on the clinical career development model of nurses in South Korea [21], which was based on Benner's "novice to expert" theory [22]. According to one study, a clinical career was defined in four-stages: novice, advanced beginner, competent, and proficient; the clinical experience associated with the proficient stage was of over 7 years [21]. Triage competency was defined by level of clinical judgment, expert assessment, management of medical resources, timely decisions, and communication based on the concept analysis study [23]. We explained the five attributes of triage competency and qualification to the nurse manager in the ED. Based on the above criteria, the nurse managers in the EDs and the research team members recommended three experts. Experience in the ED of the recommended experts was between 12 and 18 years. All experts had a master's degree and were qualified in a variety of areas, including emergency care in advanced practice nursing, basic life support (BLS), and advanced cardiac life support (ACLS).

We developed nine patient scenarios to assess concordance of the triage outcomes of the three experts. These scenarios included cardiac, cerebrovascular, and severe trauma cases which are major emergency conditions in South Korea [24]. We provided the three experts with guidelines for the evaluation of the medical records and explained the details of these guidelines. The three experts independently triaged each of the nine scenarios using KTAS, and the inter-rater agreement was assessed as ICC (intra-class correlation coefficient). The mean ICC was .92, which was excellent [25]. Finally, three recommenced experts were selected as the experts in this study.

#### Triage accuracy

The triage experts reviewed the initial nursing records and identified the true KTAS score. The experts evaluated whether KTAS score recorded by the emergency nurses in the medical records were valid. Medical records with insufficient data for the evaluation of the true triage score were excluded from the analysis. In this study, mistriage was defined as a mismatch between the KTAS score of the emergency nurses and the true KTAS score of the triage experts. Where the experts assessed the triage classification by the emergency nurses as incorrect, they recorded the reason for the triage error. We categorized the reasons for triage errors.

In addition, we examined the effect of the number of emergency patients on triage accuracy. We calculated the number of patient visits per hour over a randomly selected 20 days within the study period and then the average number of patient visits was compared based on the accuracy and inaccuracy of triage.

### Data selection

We determined the minimum sample size on the basis of a previously reported study of the minimum sample size of weighted kappa which estimated the number of samples according to the 2*k*^2^–16*k*^2^ formula, where *k* is the sequence category [26]. According to this formula, the minimum sample size was 50–400 medical records of KTAS data with 5 ordinal levels.

**Fig 2:**
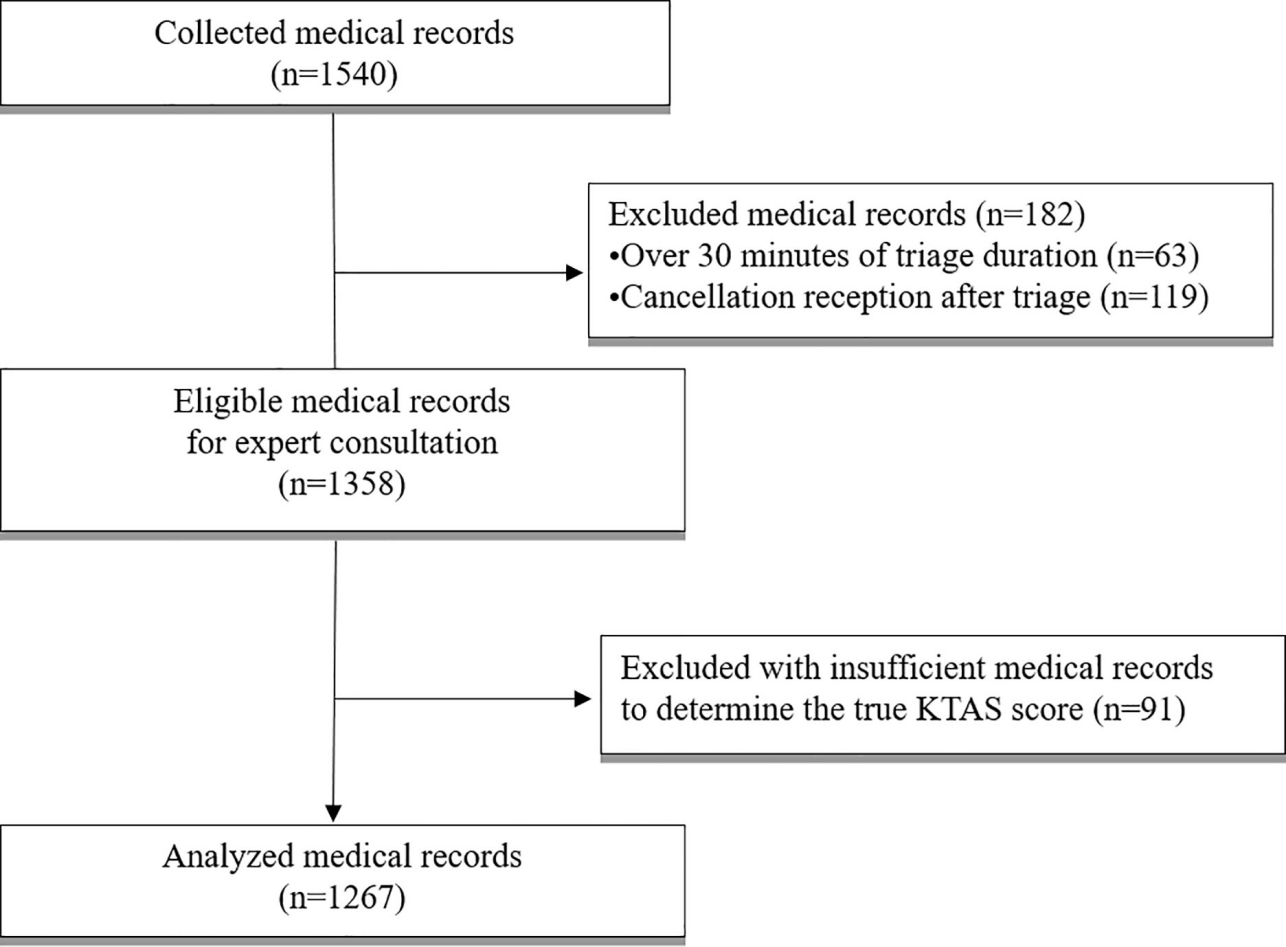
Flow chart of study process.

We randomly selected 20 days between October 1, 2016 and September 30, 2017 using a random number generator (RANDBETWEEN, Microsoft Office 365 Excel). The medical records of patients aged over 15 years old attending the 2 EDs during the selected time period were collected. A total of 1540 medical records were collected, including 676 in the regional and 864 in the local ED (Fig 2). We decided to exclude data entered after 30 minutes from the initial nurse assessment due to the possibility of revision of the original data. For instance, sometimes, the staff including doctors or senior nurses in ED provide advice regarding the triage result after completion of the initial nursing record. If the triage nurse corrects the triage score according to the staff’s advice, time of completion of the initial nursing record gets delayed. In such a case, this document would not be fit to evaluate triage accuracy due to interference from others. Therefore, the exclusion criteria for the collected medical records according to the expert advice were as follows: 1) duration of triage ≥ 30 min (n = 63), 2) cancellation at reception before triage (n = 119), and 3) incomplete data to evaluate true triage (n = 91). Finally, we included 1267 eligible medical records. The number of selected medical records was sufficient to verify the accuracy of KTAS.

### Data analysis

Data were analyzed using Stata 15.0 and SPSS (version 25.0; IBM SPSS, Armonk, NY, USA). The general characteristics of patients attending the EDs are presented as actual numbers and percentages. We compared the KTAS of emergency nurses and the true KTAS of experts using a cross table, the weighted kappa coefficient, and Pearson’s r coefficient. A *p* value < .05 was considered statistically significant in this study. An independent sample t-test was conducted to compare the number of patient visits per hour according to the accuracy and inaccuracy of triage.

### Ethical approval

This study was approved by the hospital institutional review board (IRB) (Chonnam National University Hospital; approval number: CNUH-2017-318). Medical records were anonymized so that neither the patient nor the emergency nurse could be identified. It was not possible to obtain consent from patients because the participants in this study included patients who had died or whose contact information had changed. Excluding these patients could affect the validity of the study. Therefore, we submitted a request for waiver of informed consent to the IRB Committees, which was approved.

## Results

We collected data from a total of 1267 medical records. The mean age of the participants was 54.39±19.72 years (Table 1). Overall, 753 (59.4%) patients, which was more than half, used a private vehicle. The reason for visiting the ED was related to non-injurious incidents in 1023 (80.7%) cases; however, 714 (56.4%) patients reported pain with a mean numeric rating scale (NRS) score of 4.10 ± 1.42. After treatment in the ED, 823 (65.0%) patients were discharged and 373 (29.4%) patients were admitted to the wards.

**Table 1:** Demographic data of participants.

		Count	Percentage
Age (years)	16–44	419	33.0
	45–64	399	31.5
	65–79	338	26.7
	≥80	111	8.8
	Average age (mean, SD)	54.39±19.72	54.39±19.72
Gender	Female	606	47.8
	Male	661	52.2
Type of ED	Regional ED (4th degree)	579	45.7
	Local ED (3rd degree)	668	54.3
Mode of arrival	Walking	79	6.2
	Private vehicle	753	59.4
	Public ambulance	266	21.0
	Private ambulance	155	12.2
	Other	14	1.2
Reason for visit	Injury	244	19.3
	Non-injury	1023	80.7
Underlying disease	Yes	943	74.4
	No	324	25.6
History of surgery	Yes	165	13.0
	No	1102	87.0
History of allergy	Yes	75	5.9
	No	1192	94.1
Mental states	Alert	1187	93.7
	Verbal response	39	3.1
	Pain response	28	2.2
	Unresponsive	13	1.0
Pain	Yes	714	56.4
	No	553	43.6
	Pain score by NRS (mean, SD)	4.10±1.42	4.10±1.42
Disposition	Discharge (included AMA discharge)	823	65.1
	Admission to ward	373	29.4
	Admission to ICU	8	0.6
	Transfer	32	2.5
	Surgery	22	1.7
	Death	9	0.7

ED,emergency department; NRS, numeric rating scale; KTAS, Korean Triage and Acuity Scale; ICU, intensive care unit; AMA, against medical advice

The results of the comparison of the triage accuracy between the emergency nurses and the experts were as follows. The emergency nurses and experts agreed on a triage score in 1081(85.3%) cases. The emergency nurses and experts did not agree on triage scores in 186 (14.7%) of cases. Emergency nurses showed excellent agreement with true triage results (Table 2, weighted-kappa = .829; Pearson r = .878, *p* < .001) [25].

**Table 2:** Agreement between emergency nurses and experts.

		Expert KTAS score N (row %)					Overall N (row %)
		1	2	3	4	5	
Nurses KTAS Score N (row %)	1	15(1.2)	3(0.2)	0(0.0)	0(0.0)	0(0.0)	18(1.4)
	2	11(0.9)	183(14.4)	16(1.3)	4(0.3)	0(0.0)	214(16.9)
	3	0(0.0)	27(2.1)	400(31.6)	20(1.6)	0(0.0)	447(35.3)
	4	0(0.0)	6(0.5)	63(5.0)	420(33.1)	12(0.9)	501(39.5)
	5	0(0.0)	1(0.1)	8(0.6)	15(1.2)	63(5.0)	87(6.9)
Overall N (column %)		26(2.1)	220(17.4)	487(38.4)	459(36.2)	75(5.9)	1267(100.0)

KTAS, Korean Triage and Acuity Scale

**Fig 3:**
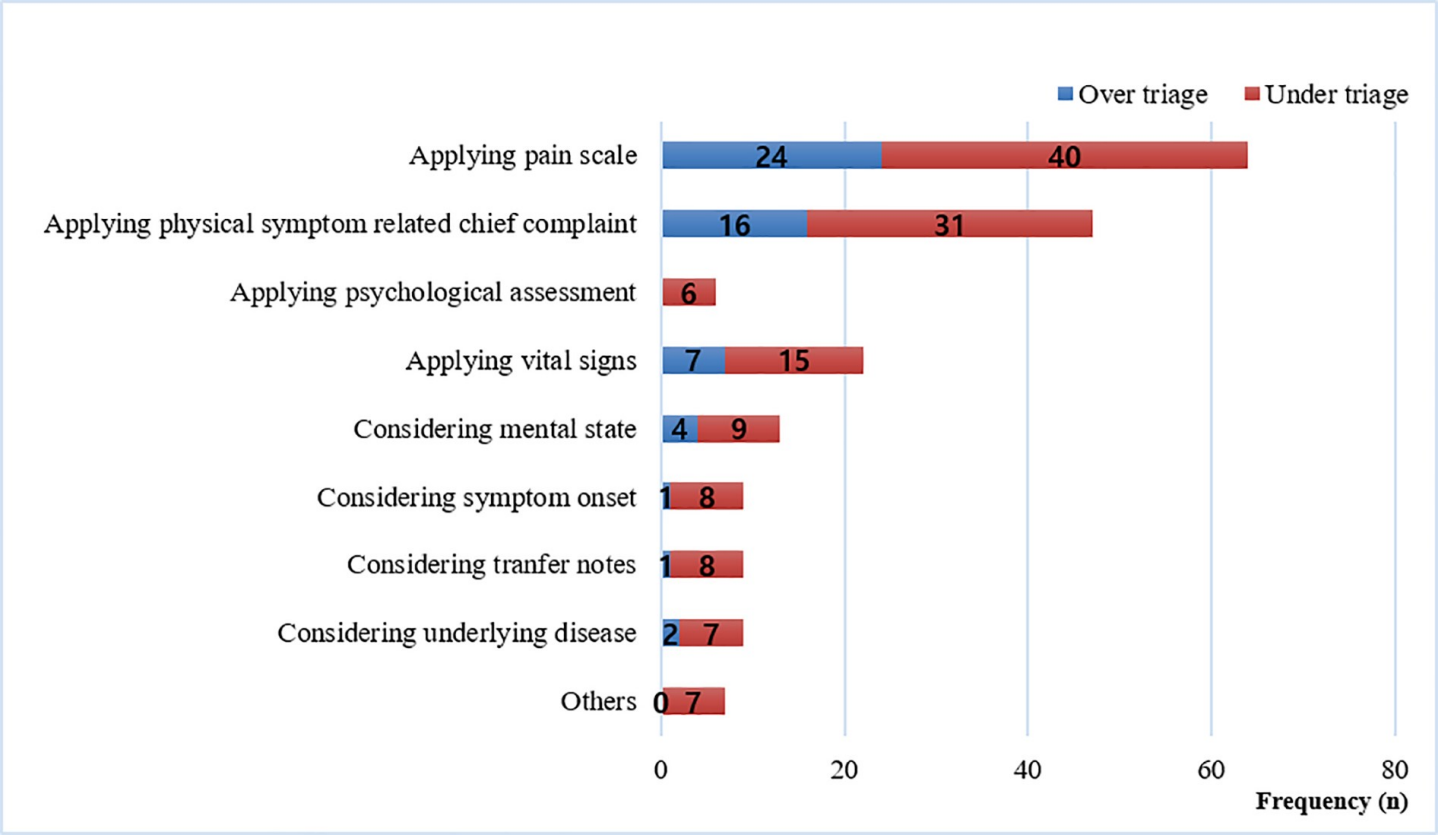
Causes of triage error (n = 186).

Among the cases classified as triage errors compared with the true KTAS score, there were 55 (29.6%) cases of over triage (overestimating the severity) and 131 (70.4%) cases of under triage (underestimating the severity). The most frequent reason for triage error was the incorrect application of the pain scale score using the KTAS criteria (Fig 3, n = 64, 34.4%). For example, the triage nurse assigned a KTAS score of 2 to a 56-year-old female patient complaining of acute abdominal pain with stable vital sign and an NRS score of 6 for pain. In this case, the experts evaluated over-triage, and according to the criteria of acute central pain experienced by patients based on body cavity or body organs; NRS 4~7 points were appropriate for KTAS 3. The second most frequent cause of mistriage was the misjudgment of physical symptoms associated with the chief complaint (n = 47, 25.3%). For example, the triage nurse assigned a KTAS score of 3 to a 70-year-old male patient complaining of diffuse chest discomfort with no radiation, presenting with diaphoresis and nausea, with stable vital signs and an NRS score of 1 for chest pain. The experts assessed this case as under triage on the basis that the patient had a suspicion of cardiogenic disease and a KTAS 2 should have been assigned requiring physician assessment within 10 minutes and an immediate electrocardiogram (ECG) check.

There was no significant difference in the number of patient visits per hour for triage accuracy (Mean ± SD = 7.55±3.14) and inaccuracy (Mean ± SD = 7.35±3.26); t = -0.77, *p* = .442.

## Discussion

Triage is an effective strategy to improve resource consumption and reduce ED overcrowding [1, 2]. There is an increasing need for accurate triage in crowded EDs. For effective triage, it is important to ensure accurate classification. This study was conducted to determine KTAS accuracy and to identify the causes of mistriage following the implementation of the KTAS in Korea in 2016 and these results can be used to improve emergency care.

Different triage tools have been developed and previous studies have evaluated triage scale accuracy. Among the major triage scales, the reliability of the CTAS has been demonstrated. In 8 studies, inter-observer reliability of the CTAS was reported to be good or excellent with a kappa value of .68-.89 [3]. The reliability study of the Taiwan Triage and Acuity Scale based on the CTAS reported comparable results, with a kappa value of .87[27]. In this study, the kappa value between the triage nurses and the triage experts was .83, showing excellent (weighted kappa = .75~1.00) inter-rater agreement based on a rule of thumb accepted in behavioral and medical sciences [25]. The reliability of the KTAS has previously been reported. In the reliability study of the KTAS by emergency nurses and medical students, the weighted-kappa was .72, which was comparable to this study [11]. In contrast, a reliability study conducted before the introduction of KTAS and examining agreement between 2 nurses reported a weighted-kappa of .39 [19]. This difference may be explained by unfamiliarity with the KTAS. The KTAS was introduced in South Korea in January 2016. In an early stage study after the introduction of KTAS, the agreement between users was low. Therefore, the increased agreement observed in this study may indicate that the KTAS is now well-established in South Korea.

Mistriage should be avoided because it threatens patient safety [6]. Under-triage is an underestimation of patient condition severity which can negatively affect patient safety and lead to death [6, 7]. The frequency of under-triage in this study was reported to be higher than that of over-triage. By underestimating severity level, under-triage may result in the delayed emergency treatment of patients. Therefore, triage education should emphasize the negative effects of mistriage, including under-triage.

The aim of this study was to analyze the causes of mistriage. The mistriage rate in this study was 14.7%, which is lower than the rate of 49% reported in a meta-analysis study that included 14 reliability studies using CTAS [28]. Previous studies have only reported on the rate of mistriage; however, we have identified the cause of triage error. The major cause of mistriage in this study was the wrong application of the pain scale. In one study, a degree of pain on KTAS resulted in overtriage and had a negative influence on the predictability [20]. Patents who had uncomplicated diseases with severe pain were considered urgent but often did not have negative outcomes. The patients with a high degree of pain assigned high acuity level on KTAS but would discharge after given analgesics (e.g. ureter stone with severe pain). Therefore, predictability of KTAS in a previous study may be the result of overtriage when compared with outcomes included emergency operation, the admission rate, the 7-day mortality [20]. In our study, the frequency of mistriage related to the pain scale was more related to under-triaged than to over-triage. These differences may be due to a disparity of focus in each study. We focused on not whether KTAS level based on pain criteria accurately predicted the medical outcomes, but whether KTAS is being applied correctly according to the criteria. Therefore, rather than making a direct comparison with previous studies, it would be more appropriate to discuss why pain was the greatest cause of mistriage by looking at the detailed pain criteria in KTAS. In KTAS, the pain scale for adults was divided into three categories: central vs peripheral, acute vs chronic and mild vs moderate vs severe [5]. Because of these complex criteria, triage nurses might find it difficult to apply pain scale results to the triage algorithm. Based on our study, the KTAS education programs should focus on the criteria of the pain scale. Another cause of mistriage in this study was wrong assessment of the physical symptoms associated with the chief complaint. Some triage nurses in this study did not concentrate on patient symptoms related to the chief complaint, the causes of which may be a lack of knowledge related to diseases, chief complaints, related symptoms and lack of experience in identifying cues based on a patient’s symptoms. Assessment of physical symptoms is one of the elements required to achieve triage competency among emergency nurses [29, 30]. Therefore, triage education will need to be further developed to strengthen structured knowledge and experience.

The methodological considerations of this study are as follows. A gold standard is required to determine the accuracy of triage. Previous studies have reported triage accuracy using inter-rate reliability in patient scenarios [10]. Scenario-based research is likely to be feasible for the study because it is easy to determine the gold standard. As this study did not use scenarios, we carefully designed the true triage to be a gold standard. We selected the experts according to strict criteria and verified the agreement among experts. The ideal triage study would involve different evaluators using the same scale in a real-world setting [13]. However, a real-world setting study can affect patient safety because it delays the patient's treatment time [13]. Therefore, we selected retrospective medical record analysis in order to evaluate triage of the actual patient while ensuring the safety of the patient.

Historically, triage was introduced to decrease ER crowding [1, 2]. However, the overcrowding environment reverses the triage process in ED. Studies based on the analysis of medical records reported that crowding had no effect on the triage destination, but had a negative effect on waiting times for triage which were longer as ED occupancy increased [31]. The present study found that the number of patient visits per hour to the EDs was not associated with triage accuracy. These findings and the results of the previous study suggest that ED overcrowding may affect the triage process, but not triage outcome.

The limitations of this study are as follows. First, this study focused on the results of decision making by emergency nurses using the medical records. We were not able to assess how the emergency nurses had reached a final decision on the classification of severity through a process of reflection. The causes of mistriage may be more clearly understood by combining the results of the decision-making processes with interviews of emergency nurses in cases of mistriage. Second, we retrospectively analyzed medical records of randomly selected dates. One limitation of this retrospective study was that we did not investigate detailed information on the patient's symptoms and signs for determining the true triage. These data have been excluded from the analysis and therefore we could not verify the impact of the excluded data on the results. Another limitation is that this study did not reflect the entire study period because of the randomly selected dates. However, we believe that this random selection did not reduce the accuracy of the results. Third, this study was conducted in two EDs and it is difficult to generalize these results. However, we found similar rates regarding admission, discharge, and death when compared with Emergency Medical Statistics in 2016 [32]. It is important to conduct nationwide studies of triage accuracy in the EDs.

## Conclusion

In this study, emergency nurses classifying severity of disease using the KTAS showed substantial concordance with experts. The major cause of triage error was inadequate application of the pain scale to the KTAS algorithm. When the triage nurse cannot correctly apply a pain scale, patient safety may be threatened by triage errors. If necessary, the pain criteria on KTAS should be simplified. Based on the results of this study, patient safety and quality of emergency medical treatment can be improved by developing an education program for the medical staff based on the cause of mistriage.

## Supporting information

S1 Appendix EMR review coding data. (XLSX)

S2 Appendix EMR review coding guideline. (XLSX)
